# Better safe than sorry – a qualitative study of multidisciplinary use of the V-RISK-10 in assessing patients with psychosis

**DOI:** 10.3389/fpsyt.2025.1561082

**Published:** 2025-09-29

**Authors:** Andreas Seierstad, Bente Kristin Bruu, Olivia Schjøtt-Pedersen, André Løvgren, Øyvind Lockertsen

**Affiliations:** ^1^ Section for Early Intervention in Psychosis, Division of Mental Health and Addiction, Oslo University Hospital, Oslo, Norway; ^2^ Institute of Clinical Medicine, University of Oslo, Oslo, Norway; ^3^ Division of Mental Health and Addiction, Oslo University Hospital, Oslo, Norway; ^4^ Faculty of Health Sciences, Oslo Metropolitan University (OsloMet), Oslo, Norway

**Keywords:** multidisciplinary teams, structured professional judgement tools, violence risk, screening, psychosis, inpatient care

## Abstract

**Background:**

Violence in mental health inpatient settings is a recognized challenge and structured professional judgement (SPJ) tools might aid clinicians in assessing risk and tailoring preventive interventions. Multidisciplinary collaboration when completing SPJ tools have been considered a feasible approach to reducing bias, increasing transparency and linking risk assessment with risk management. This study aims to explore experiences among health care professionals on multidisciplinary use of the SPJ tool violence risk screen V-RISK-10 in a closed psychosis ward.

**Methods:**

Data were collected by means of semi-structured interviews with a heterogeneous sample of health care professionals (n=8) recruited from a psychosis ward at a psychiatric hospital in Norway. Snowball sampling was utilized to recruit participants who had experiences relevant for the study’s aim. Transcribed interviews were analyzed using thematic analysis.

**Results:**

Three overarching themes were identified: 1) attitudes toward screening for possible violence, 2) attitudes towards using the V-RISK-10 and 3) attitudes toward multidisciplinary use of the V-RISK-10. In summary, subthemes revealed that screening was perceived as important, and participants perceived the tool in question as quick and systematic yet noted that missing information on the day of admittance was problematic. Important discussions were sparked and targeted interventions initiated as a result of multidisciplinary collaboration.

**Discussion:**

Staff attitudes have been described as a potential barrier for the use of SPJ tools, yet little empirical knowledge exists on the beliefs behind staff attitudes. Our study sheds light on what staff found helpful and not helpful when using the V-RISK-10. These findings can aid implementation of SPJ tools, especially screening instruments, because they highlight possible pathways toward increased use for health care professionals.

## 1 Introduction

Violence is highly prevalent in inpatient psychiatric settings, although occurrence varies greatly from ward to ward, and both fellow patients and staff fall victim to violence (1). Patients who have committed acts of aggression are often subjected to restraint and seclusion (2). Violence and aggression may also have detrimental effects on the ward atmosphere, and attaining a safe and calm ward is considered crucial to provide sound therapeutic care (2). The presence of certain psychotic symptoms, particularly command hallucinations, paranoid delusions and thought disorders, is considered predictive of inpatient violence (3). Moreover, the first days after admission has been identified as a period where most violent incidents occur (4, 5), warranting the need for an early approach to tailoring preventive interventions.

Violence risk screening tools aid clinicians in identifying patients who may be at risk of committing violence, allowing staff to quickly initiate preventive strategies (6). The structured professional judgement (SPJ) approach rely on validated tools for structured appraisal of empirically based risk factors (7). Most SPJ tools offer practitioners the opportunity to add extra risk factors and considerations as required (8). The V-RISK-10 is a violence risk screen validated for acute psychiatric inpatient settings (9, 10). The tool consists of 10 items covering historical, clinical and risk management items, followed by an overall clinical evaluation of violence risk (low/moderate/high). The clinician is also prompted to suggest whether a more detailed violence risk assessment is required, and/or preventive measures are to be implemented (10).

Although the use of validated instruments is encouraged over unstructured methods (11) clinical uptake is limited (12). Health care professionals consider risk assessment and management their responsibility, yet their attitudes toward SPJ tools vary to the extent that attitudinal change might be a prerequisite for implementation (13). Conducting multidisciplinary violence risk assessments has been put forward as a desirable path towards increasing consensus, reducing bias (14) and formulating risk management strategies following risk assessment (15). Developing risk management plans in multidisciplinary teams and putting effort into communicating them to other professionals is considered to be best practice (8). If attitudinal change is needed (13), knowledge on attitudes could be of pivotal interest. Exploring staff’s clinical decision making in relation to SPJ tools have been suggested as a relevant focal point of studies (16).

A recent, large qualitative study conducted within early intervention in psychosis treatment in the UK recommend improving violence risk assessment in these highly specialized services (17). The study found that clinicians considered baseline violence risk assessment as screening and identified no set way that risk was documented or communicated to others. The clinicians also reported time pressure issues and lacking confidence in their own clinical skills when conducting risk assessment (17). The authors recommend identification of contextually appropriate pathways toward improving collaborative violence risk assessment (17). To our knowledge, no studies have scrutinized multidisciplinary use of a SPJ screening tool as a way of improving clinical work toward violence prevention. However, a more recent risk screener for youth, the V-RISK-Y recommend that the instrument is scored interdisciplinary if feasible (18). In addition, very few studies outline staff attitudes toward SPJs in early intervention in psychosis services.

Therefore, the present study aims to investigate staff attitudes regarding multidisciplinary violence risk screening using the V-RISK-10 in a closed, early intervention in psychosis treatment ward. Furthermore, to enhance knowledge on how violence risk screening could inform risk management.

## 2 Materials and methods

### 2.1 Design and setting

This exploratively designed qualitative study was conducted in a closed psychosis treatment ward in a specialized section for early psychosis treatment at Oslo University Hospital, Oslo, Norway. The section had an inpatient ward with 11 hospital beds with a 90 day average length of stay, and an outpatient unit delivering treatment to approximately 80 patients at the same time.

According to hospital guidelines completion of the V-RISK-10 is mandatory when admitting new patients, with the admitting physician being responsible for the completion of the risk assessment. Multidisciplinary collaboration on the day of admittance is not stipulated as mandatory.

### 2.2 Intervention

To bolster staff’s clinical skills in the use of the V-RISK-10, a didactic intervention was developed by authors AS and BKB under the guidance of author ØL. Between January and March 2021 all clinical staff in the section received a one hour-long, mandatory didactic intervention on the use of V-RISK-10. In total, 4 didactic interventions were held. During the intervention participants completed the V-RISK-10 on a fictitious clinical case in multidisciplinary teams, consisting of physicians/psychologists and nurses/learning disability nurses/social workers. Participants were afterwards encouraged to utilize multidisciplinary screening with the V-RISK-10 in their clinical practice. No other didactic interventions, research activity or other possibly confounding interventions were instigated until qualitative interviews were conducted.

### 2.3 Recruitment and participants

Since the aim of this study was to explore highly specific and contextualized clinical experiences, i.e. the experiences of staff that had utilized the V-RISK-10 in a multidisciplinary setting, snowball sampling (19) was used. Author AS regularly attend clinical meetings in the ward and was able to identify a physician and a learning disability nurse who had conducted a multidisciplinary assessment with the V-RISK-10. Author BKB, who is not employed in the ward, yet know many of the participants due to her employment in the outpatient clinic, contacted individuals per e-mail and offered study participation. After completing the interviews, participants were asked if they a colleague in the ward who might also have used V-RISK-10 multidisciplinary. The process was then repeated until all staff who were mentioned as potential interviewees had been interviewed. This recruitment strategy led to the inclusion of eight (n=8) participants. Data saturation (20) was discussed between authors AS and BKB at this time, and deemed rich, illustrative and varied enough to proceed with data analysis. Participants were offered to read transcripts of their interviews. Two asked for this and were provided with a copy.

The inclusion criteria for participation in the study were the following: Being currently employed in the inpatient ward, working in a full-time position and conducting patient admittance to the ward as a part of their day to day activities, as well as being identified by another interviewee as a person that might have a view on multidisciplinary use of V-RISK-10. Thus, physicians in training and psychiatrists as well as nurses and learning disability nurses met the inclusion criteria. No specific exclusion criteria were stipulated, but since patients were solely admitted during the day, night staff were not included.

To preserve the anonymity of the participants the sample characteristics are described at group level. The sample (n=8) consisted of two physicians undergoing training to become psychiatrists, two psychiatrists, two nurses and two learning disability nurses. In the clinical setting where this study was conducted nurses and learning disability nurses perform the same clinical duties and are considered equally qualified (both professions require a bachelor’s degree and state authorization to practice clinical work). Physicians in training and psychiatrist perform the same practical duties when admitting new patients. On the day of admittance of new patients’ doctors/psychiatrists and nurses/learning disability nurses work in teams of two to perform duties such as initial clinical assessments and first interview, contact with next of kin and practicalities such as allocation of patient room. 15 nurses/learning disability nurses and three physicians performing patient admittance were employed in the ward at any given time during the study. Four physicians were interviewed because doctors in training rotate clinical placements within a timespan of 10–14 months. Thus, two physicians in training met inclusion criteria with the studies timeline. Four of the participants were female. The age range spanned between late twenties and early forties, with most being in their early thirties.

### 2.4 Data collection

An interview guide (Appendix 1) for the semi structured interviews was developed by authors AS and BKB with feedback from author ØL. The guide was developed through a reflective process between the three authors. The 8 interviews were conducted between March 2021 and March 2022, the first taking place approximately one year after the last didactic intervention. We planned to conduct the interviews 6 months after the last didactic intervention, and thus allowing interviewees the time to form their own opinions on the topic in focus. The delayed and extended timeline of interviews was due to several phases of infection control measures imposed by the Covid-19 pandemic barring contact between interviewer and interviewees. The mean time between receiving the didactic intervention and being interviewed have been calculated to 529 days, with a median of 578 days. Interviews were held in a neutral meeting room in the inpatient ward or in the outpatient clinic, due to varying accessibility of meeting rooms and lasted on average 40 minutes.

### 2.5 Data analysis

The interviews were transcribed verbatim and analyzed with thematic analysis. First, the material was read and reread to gain data familiarity. Second, data was organized into meaningful codes. Third, codes were categorized in a codebook and classified into relevant themes. Fourth, themes were reviewed by reading the codebook and rereading all interviews in order to confirm thematic validity. Fifth, themes were named and organized in a bracket system. Sixth, the report was written.

**Figure 1:**
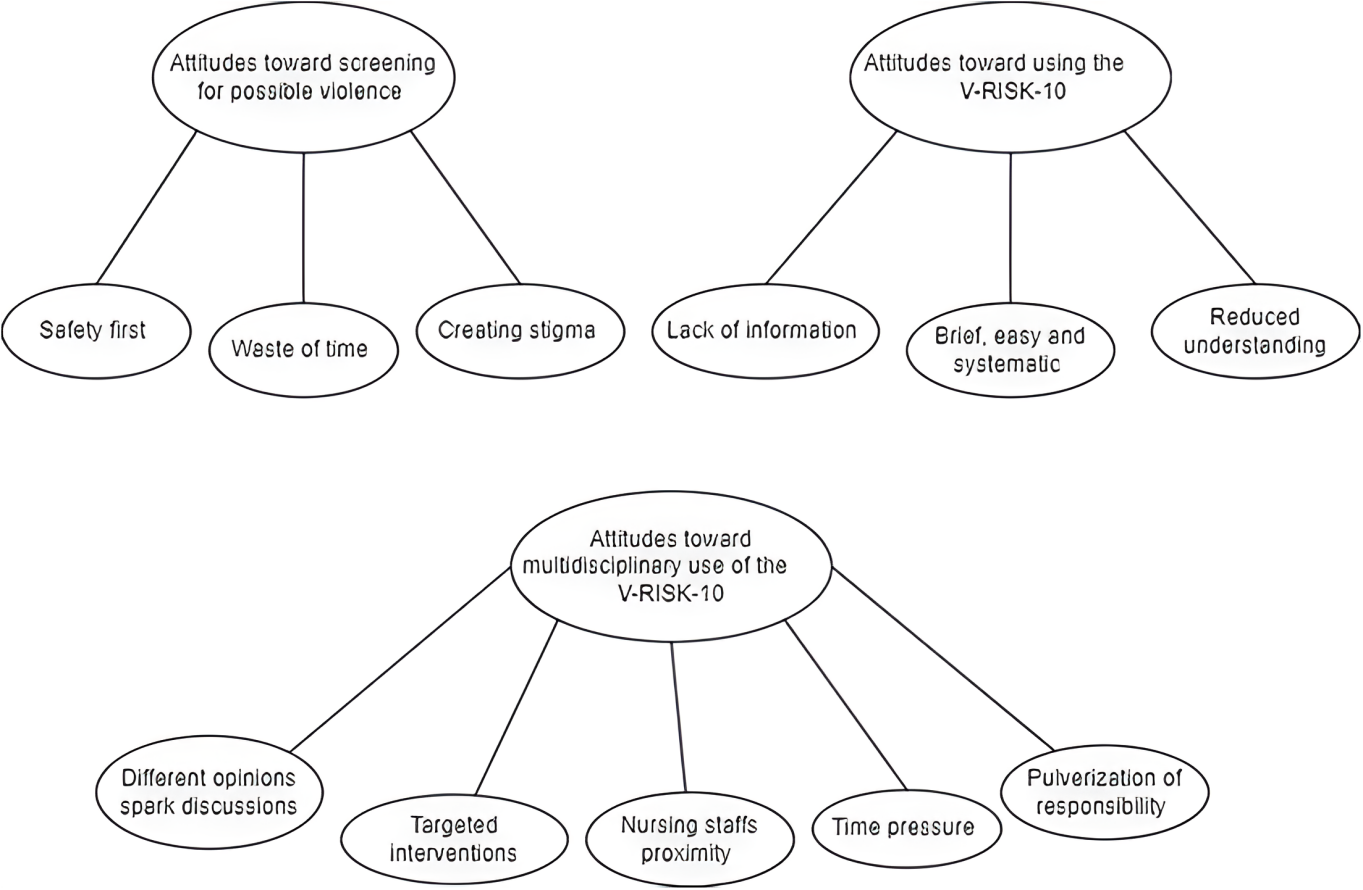
The figure is a visualization representation that show the relationship between themes and subthemes in the study.

Author AS transcribed and coded interviews, while author BKB reviewed transcripts independently. Codes and perceived themes were then discussed between the two authors. Coding, and the themes that emerged, were then discussed between authors. External independent qualitative researchers, coauthors AL and OSP, peer reviewed the themes underlying codes based on quotes in Appendix 2. AL gave supervision on coding of themes. See **Figure 1** for example of how participant quotes corroborate the creation of subthemes, and Appendix 2 for tables of themes with underlying quotes.

### 2.6 Ethical considerations

Participation was based on informed written consent. This study was approved by Oslo University Hospital’s data protection officer, identifier: 19/29242, and conducted in accordance with the principles of the Helsinki Declaration (21). Collected data was stored according to Oslo University Hospital guidelines.

This study was conducted within a small sample in a specified location. Due to issues of anonymity, participants were provided with a draft of the article before its publication and asked to read the quotes it contains and respond if they felt that manners of speech or similar rendered them identifiable.

## 3 Results

**Figure 2:**
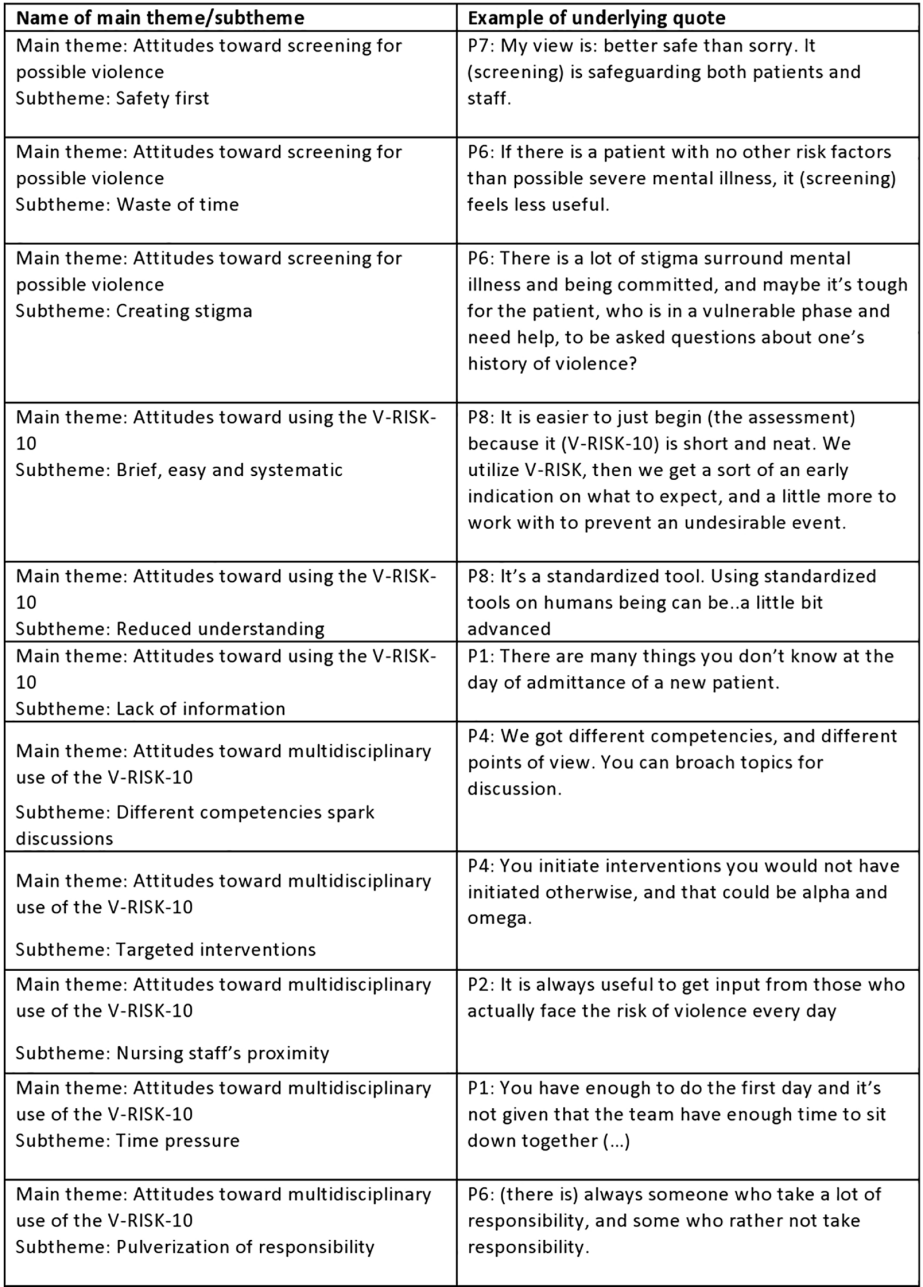
The table show the studies subthemes exemplified by an underlying quote.

Three main themes emerged: 1) attitudes towards screening for possible violence; 2) attitudes toward using the V-RISK-10; and 3) attitudes toward multidisciplinary use of the V-RISK-10. These three major themes were often described overlapping each other since participants had used the V-RISK-10 for screening purposes in a multidisciplinary setting. **Figure 2** illustrate major themes and underlying subthemes.

### 3.1 Main theme 1: attitudes toward screening for possible violence

Screening for possible violence was overall described as an important protective measure which allowed early detection of violence risk. It was noted that screening could create stigma, and also that it felt redundant when admitting patients with no apparent violence risk.

#### 3.1.1 Subtheme: safety first

The majority of participants put emphasis on mapping out violence risk as early as possible: [P7]: My view is: better safe than sorry. It (screening) is safeguarding of both patients and staff. They also noted that using V-RISK-10 as a screening instrument could help detect risk of violence at an early stage: “If we use V-RISK-10 we get an indication on what to expect, and maybe a little more to work with to avert an unwanted incident” [P3].

One of the participants also noted that discussing with others added a feeling of confidence when concluding the assessment. “It feels safe that I have discussed my thoughts with others, and it feels safe that several persons own the product together” [P5].

#### 3.1.2 Subtheme: waste of time

Three participants conveyed the notion that screening feels less useful when admitting patients with a low risk of violence. One also noted that screening might undermine violence risk assessment as a whole, since most patients who were screened ended up with a low risk of violence.

“Some patients obviously have a very low risk of committing violence, and it’s a bit like oh my god, is this what we spend our time doing? » [P2].

#### 3.1.3 Subtheme: creating stigma

Two participants described ethical concerns with violence risk screening, because they felt screening could project unfavorable preconceptions upon the patient group.

“Prejudice is a harsh word but having a preconception of a person; we need to conduct a violence risk assessment because it may be that this person is mentally ill (…) it could affect us as staff: we take it for granted that the patients may become violent” [P8].

### 3.2 Main theme 2: attitudes toward using V-RISK-10

The V-RISK-10 was overall described as a brief and systematic way of assessing violence risk, but with limitations. Concerns were voiced that a lack of information could compromise the assessment.

#### 3.2.1 Subtheme: brief, easy and systematic

Several participants described the tool as time efficient, systematical and a good reminder of important factors to consider when assessing violence risk.

P1 said: “(the VRISK-10 is) a good reminder of common risk factors for violence, which is standardized. The risk factors are mapped out, and it influences what you think about when you are writing the risk assessment. It’s important and a good reminder, and very explicit (…) You can do it quick. All forms that only consist of one page are very welcome.

#### 3.2.2 Subtheme: reduced understanding

Two of the participants reflected on that a standardized tool might deflect focus away from intrapersonal and situational factors that might have led to violence, and thus reducing a dynamic understanding of violence and its causes.

“You lose important factors, intrapersonal factors and dynamic reasoning around violence and the patient. The main challenge with it (the V-RISK-10), which is also its strength, is that it’s standardized, and you risk compromising the dynamic understanding of the patient and violence, maybe” [P1].

P8 said: It’s a standardized tool. Using standardized tools on humans can be a little bit … advanced. There is no item that taps into if there were environmental circumstances around an act of violence.

#### 3.2.3 Lack of information

Two participants noted that lacking information on the day of admittance could lead to many “do not know” answers on V-RISK-10 items. This uncertainty regarding patient factors was described as problematic. “Usually, there is many things you don’t know when admitting a new patient (…) you get lots of “don’t know” [P1].

### 3.3 Main theme 3: attitudes toward multidisciplinary use of the V-RISK-10

Multidisciplinary collaboration around completing the V-RISK-10 was described as advantageous. Participants noted that important discussions were instigated and tailored care interventions were created as part of this process. Another point was that nursing staff’s closeness to patient’s enhanced creation of care interventions. Time pressure at the day of admittance and uncertainty regarding whose responsibility it was to organize a multidisciplinary meeting was on the other hand seen as barriers to multidisciplinary collaboration.

#### 3.3.1 Subtheme: different opinions spark discussions

Participants described that the multidisciplinary team members had different opinions on a range of different topics including patient history and background [P3, P4, P7]; what variables are stronger associated with violence [P3] and what category to score on a specific item [P5]. These discussions were described as purpose- and meaningful in the sense that important topics were broached and consensus reached after all perspectives had been considered.

“Sometimes it is a bit like, how serious should we consider that episode? Does the patient meet the criteria? In those cases, I think it is useful to discuss it, I believe that assessments have been enhanced by it “[P4].

#### 3.3.2 Subtheme: targeted interventions

Several of the participants stated that multidisciplinary collaboration helped the team create individualized violence risk management interventions intended to prevent violence. It was noted that the V-RISK-10 itself incite the user to think about preventive interventions because it is mentioned at the end of the tool. However, multidisciplinary teamwork helped the team tailor strategies for individual patients, document them in the electronic nursing plan and communicate them to colleagues.

“That violence risk assessment was important, because we reviewed which interventions had been effective before (…) You can early on document in the nursing plan how one should relate to the patient in different settings (…) we had at least two staff present in the event of boundary setting, and ideally two staff present if the patient became restless or irritable (…) it was specified in the electronic treatment plan how one should talk to the patient, some need information delivered in a specific way (…)” [P8].

P4: “We assessed which room, where in the ward the patient should stay, and we moved another patient because of it (…) it was done due to violence risk, and the patient’s state of mind, the patient was hypomanic, manic as well (…) less stimuli, and in the end reduced risk of confrontation, violence and paranoia.

P6 said: “An experience from the last admittance (to the ward) was that the patient should be allowed to retreat to his own room, and that one or two nurses who knew him should accompany him; the patient should be allowed to feel in command of the situation (…) patient and staff deal with the situation according to experiences on what`s best for the patient.

#### 3.3.3 Subtheme: nursing staff’s proximity

Several of the participants noted that nursing staff work closer to the patient than other professions, and this closeness grants them access to important information about patients and knowledge about whether interventions are feasible. Participants also conveyed the sentiment that since the nursing staff is more exposed to violent behavior, it is crucial that they get a say in the initial violence risk assessment.

“Since it is the nursing staff who will handle most of the situations where the interventions will be utilized, I feel that it is important that they feel their voice is heard (…) they could also correct me if the interventions are not feasible.” [P5].

P5 said: “Because the nursing staff participate in the situations when situations arise in the ward, they have a view and an opinion on the situations which I lack: because I was not there. (…) sometimes the patient has been admitted (to the ward) before, and the nursing staff have knowledge on possible stressors in the patient’s lifestyle, domestic situation; stressful events in the past which can occur again; and which might not have been documented in a historical synopsis or psychosocial assessment.

P7 stated that “the ones working closest to the patient, the ones who spend the most time with them, in situations involving knives (i.e. cooking class), are not part of the assessment, while those who are most protected get most of the information.

#### 3.3.4 Subtheme: time pressure

Time pressure and many individual tasks associated with admitting new patients to the ward were described by several as a barrier to multidisciplinary collaboration. The planning, time and logistics required to get staff from different professions together on the day of admittance was seen as challenging: “And then there is the aspect of time; get people together for multidisciplinary assessment is the challenge. It demands more logistics (…) there is a lot to be done on their first day” [P1].

#### 3.3.5 Subtheme: pulverization of responsibility

A challenge several noted was uncertainty around whose responsibility it was to organize a multidisciplinary meeting point. Different professions had different designated tasks on the day of patient admittance, and participants described this subtheme as closely linked with time pressure. P2 said: “If several share responsibility, you get pulverization of responsibility (…) after admitting the patient I get preoccupied with my own tasks.

## 4 Discussion

The present study explored staff experiences with the V-RISK-10 in a multidisciplinary setting. The results were organized into the main themes Attitudes toward screening for possible violence, attitudes toward using V-RISK-10 and attitudes toward multidisciplinary use of the V-RISK-10, with several subthemes. In the following we will discuss some important aspects of the results.

### 4.1 Screening with the V-RISK-10: Important, but limited by missing information

Screening for violence at admittance was perceived as important. Participants highlighted that the V-RISK-10 served as a reminder of important factors relating to violence risk that the team needed to consider. This is in line with previous research where a formal risk assessment tool has been described as advantageous by staff because it allowed for collecting information in a structured manner and thus ensuring that specific areas of information were covered (15).

Participants were, however, somewhat reserved when describing the perceived usefulness of the V-RISK-10. It was noted that lacking information in the day of admittance leads to many “don’t know” scores, which was described as problematic. These reservations could be highly warranted. One recent, Norwegian study on the use of the V-RISK-10 in acute psychiatric wards found that “don’t know” scores on a range of risk factors at admission was significantly associated both with inpatient violence, as well as violence after discharge (22). The authors of the study point out that this “risk of not knowing” needs to be taken into account, and caution about considering “don’t know” as “no risk”. Their findings also underscore the need of planning violence risk management after discharge at an early point in treatment (22).

### 4.2 The burden of stigma, and the possible pitfalls of categorization

Two participants voiced concerns of stigmatizing patients by screening with the V-RISK-10. An important point to consider is that unstructured judgments of violence and their following interventions could also entail the risk of stigmatizing patients (23). However, both false positives and false negatives stemming from the use of SPJ tools can influence the care patients receive, therefore, categorizing patients with a high or low risk of violence upon admission could carry unwanted costs (24). Being labelled as potentially violent is possibly, like our participants point out, stigmatizing and it could lead to unnecessary, coercive interventions such as intense supervision or loss of liberties (24). On the other hand, interventions could prevent violence; and in effect convert true positives to false positives. Although the V-RISK-10’s has gathered support for its use in reviews (6), yet it does categorize patients and prompt interventions, with all the potentially favorable and unfavorable consequences that may follow.

### 4.3 The importance of dynamic and situational factors in violence risk assessment

Two participants commented that they felt that the V-RISK-10 is too standardized to fully assess dynamic and situational factors concerning violence, with one participant doubting its ability to tap into environmental circumstances around a violent act. Since the tool prompt users to make an overall clinical judgement based on the checklist, clinical judgement and other available information (9), one could argue that the user is well within rights to describe i.e. environmental or dynamic factors in the assessment, including interpersonal factors regarding staff members and others. Participants’ focus on the importance of dynamic violence risk assessment could be seen in light of developments in the violence risk assessment discourse. Recent areas of research have argued for the importance of considering organizational, situational and relational factors that can cause violence, instead of solely focusing on patient variables (1, 25) as well as the contribution of dynamic risk factors, i.e. symptoms that vary in intensity (26).

### 4.4 Multidisciplinary use of the V-RISK-10: The need for early instigated, but noncoercive interventions

Participants argued that early detection of potential violence risk could help treatment teams get a head start when creating interventions intended to prevent developing aggression. This is in accordance with earlier findings claiming that identifying patients with an increased risk for violence have been described as a useful application of the V-RISK-10 (6). Also, the notion that risk screening prompts interventions have been previously investigated. A study from a Nordic, non-forensic ward documented a link between high score on violence risk assessments and preventive interventions. Unfortunately the preferred methods were either coercive or pharmacological, with the authors encouraging staff to be creative when choosing risk reducing strategies (27). Over decades the focal point of risk assessment has shifted from prediction and towards prevention. In spite of this there has been a scarcity of research on how risk assessment can direct risk management (28). Our participants described a range of different interventions utilized: offering a patient a more secluded room, allowing patients to feel in command of situations, specifying communication strategies, as well as more typical strategies like added staff presence when setting boundaries. None described coercive strategies like compulsory pro re nata medication or seclusion. Therefore, it is imaginable that the participants’ beliefs stem from positive experiences with administering interventions that have deescalated conflicts instead of escalated them.

### 4.5 Assessment, intervention planning, and the role of nursing staff

Several participants described that after the multidisciplinary assessment, nursing interventions were documented in the electronic nursing plan, and it was perceived as good practice being able to present the next shift of nurses with a complete plan. This is in line with recommendations stating that effort should be put into communicating risk management plans to others (8). Also, one large study conducted on six continents found that nurses’ risk management plans were implemented more often than psychologists’ and psychiatrists’, and that nurses more often received feedback on whether their plans had been implemented (29). Moreover, psychologists reported using significantly longer time to conduct violence risk assessments than their professional counterparts (29). Hence, including nurses in assessment might save time creating violence risk management interventions even though participants described multidisciplinary collaboration as time consuming and in conflict with other clinical task, which is also in line with previous research (30). Nursing staff may also want to take part in screening due to safety issues. Participants other than nurses pointed out that the nursing staff is mostly at risk for aggressive behaviors due to their daily interactions with patients in the ward. This sentiment seems relatable: one Swedish study found that 87% of nursing staff had been exposed to inpatient violence (31).

### 4.6 Early intervention in psychosis as clinical theatre for risk assessment

The associaton between violent outcomes and schizophrenia is well documented (32) and the inpatient setting is considered especially volatile (1). For early intervention in psychosis patients violence might be especially problematic as it is correlated with hospital admittion, loss of functioning and being victimized (33–35).Thus, for young patients it might be of pivotal importance not to resort to violence. However, a recent study found that clinicians in such specific setting lack confidence in their risk assessment skills and that reporting risk to others could be challenging. The authors advocate inquiry into what could be contextually relevant pathways towards improving collaborative risk assessment (17). The present study adds on this knowledge by suggesting multidisciplinary collaboration around a brief screen could help facilitate prevention strategies and aid information flow between professionals, something especially important in a setting were violence is a primary concern (17).

### 4.7 On generalizability of findings and possible solutions to barriers of implementation

It is unclear whether our findings can be generalized to other settings. While the V-RISK-10 is considered a valid and appropriate tool for its use (6), no previous studies have to our knowledge been conducted on staff attitudes toward it in any setting. Since the tool is brief and allow for quick screening of violence and promote the use of interventions, we imagine that it could be well received in clinical settings where time is limited such as psychiatric emergency departments. We are more uncertain whether it would be seen as relevant in clinical contexts where violence is a highly relevant focus, such as forensic or security settings.

Regarding the use of risk assessment tools in general more empirical evidence exist, and our findings are in line with several sources: we found that staff were mostly in favor of using structured tools, and consider them important decision support which is in line with previous knowledge (13).We also found that different professions differ in their clinical judgement, yet found that the tool facilitated for structured information collection as documented by others (14, 15) and led to risk management interventions which also have been found elsewhere (15). Based on this we believe that our findings could be generalized to other SPJ tools in general.

Data offer no valid opportunity to assess the extent of multidisciplinary use in the ward. By applying snowball sampling we were able to identify four nursing staff (out of possible 15) and four doctors/psychiatrist that had conducted a multidisciplinary assessment with the V-RISK-10 within the study’s timeline. A limited amount of patient beds and a mean of 90 days for average length of stay could explain that proportionately more doctors had relevant experiences to share because they admit more patients each year than nursing staff. It’s also thinkable that we did not reach all possible participants because our sampling strategy relied on nursing staff telling each other how they conducted patient admittance. Other sources underscore that early intervention in psychosis (17) is a context where violence risk assessment is not a clinical priority, and our interviewees reported several reservations toward the V-RISK-10. We can assume that multidisciplinary collaboration took place but was limited in the timespan between didactic intervention and interviews. Thus, our data corroborate previous studies showing that staffs attitudes toward SPJs vary (13, 36), and might thus underscore the proposed need for attitudinal change (13) possibly through education and training (37). With this mind its thinkable that more training in the use of the V-RISK-10 could have improved utilization. A brief, 60 minute intervention can possibly offer basic training in the use of an instrument and maybe promote its use – but it’s uncertain whether it can lead to attitudinal change. It’s possible that arranging booster training session regularly for staff or providing selected employees with more extensive training in the tool, or coaching teams during clinical work might have improved uptake. Another possible facilitator for multidisciplinary use could be changing organization policy. As noted earlier, completing the V-RISK-10 is mandatory for doctors in training/psychiatrists at the ward, but involving nursing staff is not mandatory. This could explain participants view that responsibility for multidisciplinary collaboration was pulverized as responsibility was not placed by management. One could also speculate if this could create a form of role ambiguity – it’s clearly the physician ‘s responsibility to complete the tool, but who is responsible for facilitation a multidisciplinary meeting? Thus, a change of clinical recommendations or organizational policy could lead to increased use. Time pressure was also noted as a barrier, yet one could imagine that adjusting policy could lead to different clinical prioritizations.

## 5 Conclusion

If we assume that attitudes could be a barrier for the use of SPJ tools and that attitudinal change is a prerequisite for implementation, our study might indicate a path toward increased utilization. Participants described screening as important and the V-RISK-10 favorably, yet they also voiced reservations regarding missing information, the risk of stigmatization, false negatives and the risk of focusing insufficiently on dynamic factors when assessing violence risk. Multidisciplinary collaboration around the V-RISK-10 was seen as overall positive. The instigation of important discussions and tailoring of risk management interventions were highlighted. It is possible that collaboration around the tool added perceived user value to the V-RISK-10 in the sense that it increased perceived utility. A viable path toward more favorable attitudes toward SPJ tools might therefore lie in increased collaboration, and this could be a focal point for future studies. Although all participants were in favor of multidisciplinary collaboration, they also described that time pressure and unclear expectations regarding whose responsibility it was to arrange the team meeting were dominant barriers to conducting the assessment in a team. It is possible that more training in the tool and adjustments in organizational policy could facilitate for increased multidisciplinary collaboration.

### 5.1 Limitations

The major strength of this study is that it generates qualitative knowledge on an understudied theme: staff attitudes toward multidisciplinary utilization of a SPJ screening tool. To our knowledge this is the first study that illuminates the topic, and our data is rich and illustrative.

Several limitations should be observed. The sample size is limited, and although we believe that data saturation was reached, we cannot know if a larger sample size would have provided richer and more detailed data. Snowball sampling could also be a limitation, as the first two participants were chosen at the discretion of author AS, and because those who were recruited were aware of them being selected by others: this might have influenced their responses.

The main limitation we observe is that the study is conducted within the confines of the first and second authors’ workplace, and this proximity to the interview subjects could have influenced the interviews. Although participants were perceived as nuanced and candid by the interviewer, we must acknowledge that they might have been affected by social desirability bias (38). The interviewer, author BKB, was part of the intervention delivery team, and this could have incited interviewees to present views they assumed she wanted to hear. The intervention in itself may also have influenced participants to develop a more favorable view of multidisciplinary violence risk assessments with V-RISK-10. Also, this study lacks the patient’s perspective. Patients self-risk assessments can have similar predictive power as assessments by clinicians (39) and could be a viable path toward more user involved risk assessments.

## Data Availability

The original contributions presented in the study are included in the article/**Supplementary Material**. Further inquiries can be directed to the corresponding author.
