# Supplementary material for: Better safe than sorry – a qualitative study of multidisciplinary use of the V-RISK-10 in assessing patients with psychosis
Source: Front Psychiatry. 2025 Sep 29;16:1561082. doi: 10.3389/fpsyt.2025.1561082 (PMC12515802; doi:10.3389/fpsyt.2025.1561082)
Supplement: Supplementary file 1 [file Table1.docx]

Supplementary Material

# Appendix 1: Interview guide

-Can you tell me about the first time you used V-RISK-10 in your current job?

-Can you tell me about your experience with using the V-RISK-10 in your current job?

-Have you used other instruments to assess violence risk? In your experience, what is the difference between the instruments?

-What do you perceive as pros and cons of using structured violence risk tools?

-What do you perceive as pros and cons of screening all patients for violence upon admittance to the unit?

-What are your thoughts on pros and cons of conducting violence risk screening with the V-RISK-10 in multidisciplinary teams?

-In what way do you allow others to contribute to your violence risk assessments?

-In what way do you contribute to other professionals’ violence risk assessments?

-Do you have any thoughts on the training in using V-RISK-10 the section provided?

-Do you have any thoughts on your own competence regarding violence risk assessments?
