# Supplementary material for: Better safe than sorry – a qualitative study of multidisciplinary use of the V-RISK-10 in assessing patients with psychosis
Source: Front Psychiatry. 2025 Sep 29;16:1561082. doi: 10.3389/fpsyt.2025.1561082 (PMC12515802; doi:10.3389/fpsyt.2025.1561082)
Supplement: Supplementary file 2 [file Table2.docx]

Supplementary Material

# Supplementary Data

**Appendix 2: Themes with underlying quotes**

| Main theme: Attitudes toward screening for violence  Subtheme: Safety first |
| --- |
| P3: If we use V-RISK-10 we get an indication on what to expect, and maybe a little more to work with to avert an unwanted incident (…) In summary, I believe it’s positive that we focus on safety, and doing a V-RISK is a great start when admitting a new patient.  P5: It feels safe that I have discussed my thoughts with others, and it feels safe that several persons own the product together.  P7: My view is: better safe than sorry. It (screening) is safeguarding of both patients and staff.  P4: It (screening) is important because we need to map out and identify risk.  P2: I think it (screening) is important. (…) Violence risk and suicide risk are something you cannot not assess.  P6: I think it is advantageous to be conscious of it (violence risk) right away |

| Main theme: Attitudes toward screening for possible violence  Subtheme: Creating stigma |
| --- |
| P8: Prejudice is a harsh word but having a preconception of a person; we need to conduct a violence risk assessment for it may be that this person is mentally ill (…) it could affect us as staff that we take it for granted that the patients may become violent.  H: There is a lot of stigma surround mental illness and being committed, and maybe it’s tough for the patient, who is in a vulnerable phase and need help, to be asked questions about one’s history of violence? |

| Main theme: Attitudes toward screening for possible violence  Subtheme: Waste of time |
| --- |
| P2: Some patients obviously have a very low risk of committing violence, and you, it’s a bit like oh my god, is this what we spend our time doing?  P1: It (screening) was especially important to do in the intensive care unit. (…) It (V-RISK-10) feels a lot more important when you get (admitted) an amphetamine-induced psychosis in restraints accompanied by the police.  P6: If there is a patient with no other risk factors than possible severe mental illness, it (screening) feels less useful. |

| Main theme: Attitudes toward using the V-RISK-10  Subtheme: Brief, easy and systematic |
| --- |
| P8: It is easier to just begin (the assessment) because it (V-RISK-10) is short and neat. We utilize V-RISK, then we got a sort of an early indication on what to expect, and a little more to work with to prevent an undesirable event. When you’ve conducted a V-RISK you can see some contexts and maybe get and understanding of what might trigger violence or violent behavior and understand better what to look for when admitting new patients.  P2: It (V-RISK-10) doesn’t require much time.  P6: I think it is safer for me to have an instrument that needs to be brief enough that it does not feel like an extra big task to do. It is easier to overlook elements when you are not using V-RISK, I think.  P4: It is a short process doing a V-RISK-10, and you need to condense it (the assessment).  P5: The best part is that you think about everything, all ten factors or what it is. (…) When you think about those factors, future stress and so on, it’s a good reminder. V-RISK-10 is a lot more time efficient than HCR-20.  P1: (the V-RISK-10 is) a good reminder of common risk factors for violence, which is standardized. The risk factors are mapped out, and it influences what you think about when you are writing the risk assessment. It’s important and a good reminder, and very explicit (…) You can do it quick. All forms that only consist of one page are very welcome. |

| Main theme: Attitudes toward using the V-RISK-10  Subtheme: Reduced understanding |
| --- |
| P8: It’s a standardized tool. Using standardized tools on humans being can be.a little bit advanced. There is no item that taps into if there were environmental circumstances around an act of violence.  P1: You lose important factors, intrapersonal factors and dynamic reasoning around violence and the patient. (…) the main challenge, which is also a strength, is that is standardized; maybe you understand the patient less and try less to get that dynamic understanding of violence and the patient. |

| Main theme: Attitudes toward using the V-RISK-10  Subtheme: Lack of information |
| --- |
| P1: There are many things you don’t know at the day of admittance of a new patient.  P2: Usually, there are many things you don’t know when admitting a new patient (…) you get lots of “don’t know”. |

| Main theme: Attitudes toward multidisciplinary use of the V-RISK-10 Subtheme: Different competencies spark discussions |
| --- |
| P5: The physicians got their competency, and the nurses and social educators got their competency.  P4: We got different competencies, and different points of view. You can broach topics for discussion.  P8: Professions or people with different backgrounds can notice different aspects (…) I noticed that people were not afraid to voice their opinions.  P6: Sometimes it is a bit like how serious should we consider that episode? Does the patient meet the criteria? In those cases, I think it is useful to discuss it, I believe that assessments have been enhanced by it. |

| Main theme: Attitudes toward multidisciplinary use of the V-RISK-10 Subtheme: Targeted interventions |
| --- |
| P1: V-RISK-10 can quickly become one of those tic- tic-the-boxes-exercises on statistical factors that predict violence, but what really matters is stated at the end of the form; regarding interventions.  P4: You initiate interventions you would not have initiated otherwise, and that could be alpha and omega. It (V-RISK-10) forces you to do it systematically. (…) You initiate interventions rapidly because you’re forced to think about it. (…) you work differently, then you think differently, you think about interventions: early interventions (…). We assessed which room, where in the ward the patient should live, and we moved another patient because of it (…) it was done due to violence risk and the patient’s state of mind, the patient was hypomanic, manic as well (…) less stimuli, and in the end reduced risk of confrontation, violence and paranoia.  P5: What is best about it (multidisciplinary collaboration) is deciding on interventions together.  P8: “That violence risk assessment was important, because we reviewed which interventions had been effective before (…). You can early on document in the nursing plan how one should relate to the patient in different settings (…) we had at least two staff present in the event of boundary setting, and ideally two staff present if the patient became restless or irritable (…) it was specified in the electronic treatment plan how one should talk to the patient, some need information delivered in a specific way (…)”.  P2: You could get input on interventions; what is sensible to do.  P6: An experience from the last admittance (to the ward) was that the patient should be allowed to retreat to his own room, and that one or two nurses who knew him should accompany him; the patient should be allowed to feel in command of the situation (…) patient and staff deal with the situation according to experiences on what`s best for the patient. |

| Main theme: Attitudes toward multidisciplinary use of the V-RISK-10  Subtheme: Nursing staff’s proximity |
| --- |
| P2: It is always useful to get input from those who actually face the risk of violence every day.  P1: I think it is advantageous to sit down together and discuss interventions. It is the nursing team who is with patients twenty-four seven and have to deal with violence.  P5: Since it is the nursing staff, who will handle most of the situations where the interventions will be utilized, I feel that it is important that they feel their voice is heard (…) they could also correct me if the interventions are not feasible.  P7: I am not certain that the assessment itself would be different if it was conducted multidisciplinary, but the information discussed in that setting is relevant for everyone in a team (…) those who work closest to the patients, spend a lot of time alone with patients, outdoors, in situations with knives (i.e. cooking class) don’t participate in the assessment, while those who are most protected might get the majority of the information.  P5: Because the nursing staff participate in the situations when situations arise in the ward, they have a view and an opinion on the situations which I lack: because I was not there. (…) sometimes the patient has been admitted (to the ward) before, and the nursing staff have knowledge on possible stressors in the patient’s lifestyle, domestic situation; stressful events in the past who can occur again; and who might not have been documented in a historical synopsis or psychosocial assessment. |

| Main theme: Attitudes toward multidisciplinary use of the V-RISK-10  Subtheme: Time pressure |
| --- |
| P7: There is a lot to do the first day.  P1: You have enough to do the first day and it’s not given that the team have enough time to sit down together and go through all those items (of the V-RISK-10) because there is one thousand other things to do. |

| Main theme: Attitudes toward multidisciplinary use of the V-RISK-10  Subtheme: Pulverization of responsibility |
| --- |
| P2: If several share responsibility the, you get pulverization of responsibility. (…) after admitting the patient I get preoccupied with my own tasks.  P6: (there is) always someone who take a lot of responsibility, and some who rather not take responsibility. |
