# Supplementary material for: Better safe than sorry – a qualitative study of multidisciplinary use of the V-RISK-10 in assessing patients with psychosis
Source: Front Psychiatry. 2025 Sep 29;16:1561082. doi: 10.3389/fpsyt.2025.1561082 (PMC12515802; doi:10.3389/fpsyt.2025.1561082)
Supplement: Supplementary file 3 [file DataSheet1.pdf]

Violence risk screening -10 (V-RISK-10)

At admission ☐
At discharge ☐
In policlinic ☐

Patient's name:		Date of birth:
Female ☐ Male ☐		Patient number:
Date of admittance:	Date of discharge:	Registration number:
Signed in by:		Date:

Scoring instruction:

The rater collects information about each of the ten risk factors on the V-RISK-10 checklist. Some examples of important scoring information are described under each item. Put a check in the box to indicate the degree of likelihood that the risk factor applies to the patient in question:

- **No:** Does not apply to this patient
- **Maybe/moderate:** Maybe applies/present to a moderately severe degree
- **Yes:** Definitely applies to a severe degree
- **Do not know:** Too little information to answer

Item	No	Maybe/ moderate	Yes	Do not know
**1. Previous and/or current violence**<br>*Severe violence refers to physical attack (including with various weapons) towards another individual with intent to inflict severe physical harm.* ***Yes:*** *The individual in question must have committed at least 3 moderately violent aggressive acts or 1 severe violent act. Moderate or less severe aggressive acts such as kicks, blows and shoving that does not cause severe harm to the victim is rated* ***Maybe/moderate.***	☐	☐	☐	☐
**2. Previous and /or current threats (verbal/physical)**<br>***Verbal:*** *Statements, yelling and the like, that involve threat of inflicting other individuals physical harm.*<br>***Physical:*** *Movements and gestures that warn physical attack.*	☐	☐	☐	☐
**3. Previous and/or current substance abuse**<br>*The patient has a history of abusing alcohol, medication and/or other substances (e.g. amphetamine, heroin, cannabis). Abuse of solvents or glue should be included. To rate* ***Yes****, the patient must have and/or have had extensive abuse/dependence, with reduced occupational or educational functioning, reduced health and/or reduced participation in leisure activities.*	☐	☐	☐	☐
**4. Previous and/or current major mental illness**<br>***NB:*** *Whether the patient has or has had a psychotic disorder (e.g. schizophrenia, delusional disorder, psychotic affective disorder).*<br>*See item 5 to rate personality disorders.*	☐	☐	☐	☐

© Centre for Research and Education in Forensic Psychiatry, Oslo, Norway
2007 Hartvig, Østberg, Alfarnes, Moger, Skjønberg & Bjørkly

	No	Maybe/ moderate	Yes	Do not know
**5. Personality disorder**<br>*Of interest here are eccentric (schizoid, paranoid) and impulsive, uninhibited (emotionally unstable, antisocial) types.*	☐	☐	☐	☐
**6. Shows lack of insight into illness and/or behaviour**<br>*This refers to the degree to which the patient lacks insight in his/her mental illness, with regard to for instance need of medication, social consequences or behaviour related to illness or personality disorder.*	☐	☐	☐	☐
**7. Expresses suspicion**<br>*The patient expresses suspicion towards other individuals either verbally or nonverbally. The person in question appears to be "on guard" towards the environment.*	☐	☐	☐	☐
**8. Shows lack of empathy**<br>*The patient appears emotionally cold and without sensitivity towards others' thoughts or emotional situation.*	☐	☐	☐	☐
**9. Unrealistic planning**<br>*This assesses to which degree the patient him/herself has unrealistic plans for the future (inside or outside the inpatient unit). Is for instance the patient him/herself realistic with regard to what he/she can expect of support from family and of professional and social network? It is important to assess whether the patient is cooperative and motivated with regard to following plans.*	☐	☐	☐	☐
**10. Future stress-situations**<br>*This evaluates the possibility that the patient may be exposed to stress and stressful situations in the future and his/her ability to cope with stress. For example (in and outside inpatient unit): reduced ability to tolerate boundaries, physical proximity to possible victims of violence, substance use, homelessness, spending time in violent environment/association with violent environment, easy access to weapons etc.*	☐	☐	☐	☐

Overall clinical evaluation

· Based on clinical judgement, other available information and the checklist:

· How great do you think the violence risk is for this patient? ***(Put a check in one of the boxes)***

LOW	MODERATE	HIGH

· Suggestion following overall clinical evaluation: ***(Put a check in one of the boxes)***

NO MORE DETAILED VIOLENCE RISK ASSESSMENT	MORE DETAILED VIOLENCE RISK ASSESSMENT

IMPLEMENTATION OF PREVENTIVE MEASURES

Justifications/reasons/arguments should be detailed in patient record and/or discharge summary

© Centre for Research and Education in Forensic Psychiatry, Oslo, Norway
2007 Hartvig, Østberg, Alfarnes, Moger, Skjønberg & Bjørkly
